# Microenvironmental regulation of T-cells in pulmonary hypertension

**DOI:** 10.3389/fimmu.2023.1223122

**Published:** 2023-07-11

**Authors:** Lydie Plecitá-Hlavatá, Andrea Brázdová, Monika Křivonosková, Cheng-Jun Hu, Tzu Phang, Jan Tauber, Min Li, Hui Zhang, Konrad Hoetzenecker, Slaven Crnkovic, Grazyna Kwapiszewska, Kurt R. Stenmark

**Affiliations:** ^1^ Laboratory of Pancreatic Islet Research, Institute of Physiology, Czech Academy of Sciences, Prague, Czechia; ^2^ Institute of Organic Chemistry and Biochemistry, Czech Academy of Sciences, Prague, Czechia; ^3^ Department of Genetics and Microbiology, Faculty of Science, Charles University, Prague, Czechia; ^4^ Department of Cell Biology, Faculty of Science, Charles University, Prague, Czechia; ^5^ Department of Craniofacial Biology School of Dental Medicine, University of Colorado, Aurora, CO, United States; ^6^ Developmental Lung Biology and Cardiovascular Pulmonary Research Laboratories, Departments of Pediatrics and Medicine, University of Colorado, Aurora, CO, United States; ^7^ Laboratory of Mitochondrial Physiology, Institute of Physiology, Czech Academy of Sciences, Prague, Czechia; ^8^ Department of Thoracic Surgery, Medical University of Vienna, Graz, Austria; ^9^ Otto Loewi Research Center, Ludwig Boltzmann Institute for Lung Vascular Research, Graz, Austria; ^10^ Institute for Lung Health, Member of the German Lung Center, Giessen, Germany

**Keywords:** pulmonary fibroblasts, HDAC inhibitors, pulmonary hypertension, T-cells, γδ T-cells, Tregs

## Abstract

**Introduction:**

In pulmonary hypertension (PH), pulmonary arterial remodeling is often accompanied by perivascular inflammation. The inflammation is characterized by the accumulation of activated macrophages and lymphocytes within the adventitial stroma, which is comprised primarily of fibroblasts. The well-known ability of fibroblasts to secrete interleukins and chemokines has previously been implicated as contributing to this tissue-specific inflammation in PH vessels. We were interested if pulmonary fibroblasts from PH arteries contribute to microenvironmental changes that could activate and polarize T-cells in PH.

**Methods:**

We used single-cell RNA sequencing of intact bovine distal pulmonary arteries (dPAs) from PH and control animals and flow cytometry, mRNA expression analysis, and respirometry analysis of blood-derived bovine/human T-cells exposed to conditioned media obtained from pulmonary fibroblasts of PH/control animals and IPAH/control patients (CM-(h)PH Fibs vs CM-(h)CO Fibs).

**Results:**

Single-cell RNA sequencing of intact bovine dPAs from PH and control animals revealed a pro-inflammatory phenotype of CD4+ T-cells and simultaneous absence of regulatory T-cells (FoxP3+ Tregs). By exposing T-cells to CM-(h)PH Fibs we stimulated their proinflammatory differentiation documented by increased IFNγ and decreased IL4, IL10, and TGFβ mRNA and protein expression. Interestingly, we demonstrated a reduction in the number of suppressive T-cell subsets, i.e., human/bovine Tregs and bovine γδ T-cells treated with CM-(h)PH-Fibs. We also noted inhibition of anti-inflammatory cytokine expression (IL10, TGFβ, IL4). Pro-inflammatory polarization of bovine T-cells exposed to CM-PH Fibs correlated with metabolic shift to glycolysis and lactate production with increased prooxidant intracellular status as well as increased proliferation of T-cells. To determine whether metabolic reprogramming of PH-Fibs was directly contributing to the effects of PH-Fibs conditioned media on T-cell polarization, we treated PH-Fibs with the HDAC inhibitor SAHA, which was previously shown to normalize metabolic status and examined the effects of the conditioned media. We observed significant suppression of inflammatory polarization associated with decreased T-cell proliferation and recovery of mitochondrial energy metabolism.

**Conclusion:**

This study demonstrates how the pulmonary fibroblast-derived microenvironment can activate and differentiate T-cells to trigger local inflammation, which is part of the vascular wall remodeling process in PH.

## 1 Introduction

Pulmonary hypertension is characterized by significant vascular remodeling and persistent inflammation, i.e. altered immune profile (1–3). Inflammatory cells, including monocyte, macrophages, dendritic cells, and various types of lymphocytes are found primarily in the adventitial region of the diseased vessel wall (4, 5). The primary stromal cell in the adventitia is the fibroblast. This raises the possibility of significant fibroblast-immune cell cross-talk in disease pathogenesis and is consistent with the observations in other diseases and organs where fibroblasts interacting with immune cells, play an important role (6, 7). Clearly, fibroblasts are now recognized to play a multifaceted role in health and disease, including being key immune “sentinel” cells (6, 8, 9). Indeed, it is now accepted by many that fibroblasts can activate and modulate immune responses and they are now acknowledged as a non-classical branch of the innate immune system. Recent studies support the idea that there are two principal fibroblast phenotypes that can be observed in tissues, both normal and diseased; immune interacting- and tissue remodeling phenotypes (10, 11). Our group has reported that we can consistently isolate in culture an adventitial fibroblast from the pulmonary hypertensive vessel wall that meets the criteria for being an immune interacting phenotype (12–17). Previous studies showed that at least some fibroblasts from the PH vessel wall, as opposed to those derived from the control vessel wall, have the ability to polarize macrophages into a very distinct pro-inflammatory, pro-remodeling phenotype (18). These observations clearly support the idea that a local microenvironment could be created in the adventitial regions of the vessel wall in pulmonary hypertension to support acute as well as potentially chronic inflammatory changes that comprise immune cells in addition to macrophages.

Data from other organs suggest the possibility that activated fibroblasts can not only act on cells of the innate immune system but also on cells of the adaptive immune system (19). Observational studies have reported a variety of T-cell subsets as well as dendritic cells in the adventitia of pulmonary hypertensive vessel walls. It has been reported that an altered CD4+/CD8+ ratio contributes to pulmonary hypertension (PH) pathology by causing abnormal vascular remodeling of the pulmonary vasculature indicating an involvement of the T-cells in PH (20). Again, it has been established that T-helper cells, in particular Th1 and Th17 induce an inflammatory response through the production of interleukin-6 (IL6), IL2, IL21, interferon-gamma (IFNγ), and tumor necrosis factor (TNFα) in PH (21, 22). The effect of these T-helper cells can be exacerbated by Th 2 dysregulation depending on IL4 and IL13 production. It is also increasingly clear that for currently unknown reasons, there is a deficiency in the T-regulatory cell population (Tregs) in the hypertensive vessel wall (23). Importantly, because these Tregs act to limit the immune response in healthy humans, the T-regulatory population accounts for approximately 5-10% of peripheral CD4+ T-cells (24). FoxP3 Tregs exhibit the strongest immunosuppressant functions (23). Their ability to secrete IL10 and TGF-α enables them to inhibit the proliferation of immune-associated cells including CD4+, CD8+ T-cells, NK, and antigen-presenting cells. An imbalance in the Treg/Th17 ratio has been described to affect the progression of PH and also to correlate with the severity and prognosis of PH (25, 26).

Collectively, these observations stimulated us to examine the effects of activated fibroblast from the PH vessel wall on T-cell subset numbers and activation status. We believe that this is important to gain insight into the entire immune dysfunction in the adventitial microenvironment that is associated with PH. It is possible that these insights can inform regarding how interventions between fibroblast immune cell interactions might become a useful target for ameliorating inflammatory responses in PH.

## 2 Materials and methods

Unless specified otherwise, chemicals were purchased from Merck Life Science, Darmstadt, Germany.

### 2.1 Cell cultures and derived media

Bovine pulmonary artery adventitial fibroblasts were isolated from control (CO Fibs) and hypoxic hypertensive PH Fibs calves as previously described (27). Isolated fibroblasts were cultured at 37°C in humidified air with 5% CO_2_ in DMEM medium (Life Technologies, Carlsbad, CA) without glucose and supplemented with 4mM glutamine, 1mM sodium pyruvate, 25mM HEPES, 10% gamma-irradiated bovine calf serum (CBS, GemCell ≤18 months old, Gemini, New York, US, irradiation 25-35 kGy), nonessential amino acids, and 25mM glucose. Experiments were performed between 4-8 passages. Media after 24 hrs of cultivation were collected for further calf T-cell cultivation/assays and referred to as conditioned media (CM), either CM-CO Fibs derived from CO Fibs cultivation or CM-PH Fibs derived from PH Fibs cultivation. To avoid rapid activation and burst of bovine T-cells isolated from calf blood, we optimized media in 3:1 ratio of RPMI: CM-CO/PH Fibs media, and approved that this media sufficiently activated T-cells within 6 (early)/17-24 (late) hrs without significant loss of viability (not shown). In case of HDAC inhibitor, SAHA, the cultivation of CO/PH Fibs was performed for 72 hrs. Collected media were referred CM-CO/PH Fibs+SAHA. Medium from human adventitial fibroblasts was delivered from donors and IPAH patients (CM-hCO/PH Fibs). IPAH patients were 2 females, aged 27/34 years, mPAP was 40-60 mmHg and donors were 2 males, aged 27/34 years. Human adventitial fibroblasts (1-3 passage) were cultured for 2-3 days to 50-80% of confluence in complete VascuLife SMC complete medium.

### 2.2 T-cell isolation

Venous blood was collected from 5-6-month-old female Holstein calves (kindly provided by the Animal Production Center of the Czech University of Agriculture in Prague, Nové Strašecí and Ruda, Czech Republic) in K-EDTA tubes. Peripheral blood mononuclear cells (PBMCs) were isolated from fresh blood by Ficoll-Paque plus density gradient centrifugation in 50 ml Falcon tubes (Corning, New York, US). Buffy coats from healthy individuals were obtained from the Institute of Hematology and Blood Transfusion (IHBT, Prague, Czech Republic). Informed written consent was obtained from each individual enrolled. The study was approved by the institutional review board of IHBT, with evidence number 13/06/2012. PBMC were isolated from buffy coats using SepMate™-50 (Stem Cell Technologies), based on Ficoll^®^ Paque Plus (GE Healthcare) density gradient centrifugation, according to the manufacturer’s protocol. Red blood cells were lysed in RBC lysis buffer (Abcam, Cambridge, UK) according to the manufacturer’s protocol. 10.10^6^ PMBC cells were seeded in 50 ml RPMI1640 medium supplemented with 10% (v/v) gamma-irradiated CBS (for bovine PBMC) and FBS (for human PBMC) and 1% (v/v) penicillin-streptomycin at 37°C and 5% CO_2_ atmosphere (referred to as standard culture conditions). Monocytes were separated from PBMC by plastic adhesion (28), and the remaining T-cell-containing fraction was collected and washed with PBS/EDTA. CD4+ T-cells were isolated with a mouse anti-bovine CD4+ antibody (clone CC30, BioRad, Hercules, CA) and rat anti-mouse IgG1 microbeads using the MiDiMACS separation kit (Miltenyi, Auburn, CA). Cytometric analysis revealed that the calf T-cell population contained 24.8% ± 6.37 CD4+ T-cells (not shown). The purity of the CD4+ T-cell population was determined by immunophenotyping analysis as follows: CD3+ CD4+ T-cells in the viable subset (Zombie NIR), which averaged 89 ± 8,6% (CD3+) and 58.8± 4,9% (CD3+CD4+), respectively. As a positive control of T-cell activation towards proinflammatory polarization LPS (6 hrs) together with nigericin (last 2 hrs) were used.

We observed a similar amount of CD4+CD25+ human T-cells under all cultivation conditions, i.e. 65.6±1.06 positive human T-cells.

### 2.3 Respirometry analysis

Oxygen consumption and acidification of the media were determined after 24 hrs of T-cell cultivation in CM-CO/PH Fibs media using the Seahorse XF24 analyzer (Agilent, Santa Clara, CA) or the high-resolution Oxygraph 2k (Oroboros, Innsbruck, Austria). The Oxygraph 2k was first calibrated in air and background corrected with a culture medium. Endogenous respiration was followed by the addition of glucose, oligomycin (ATP synthase inhibitor), FCCP/carbonyl cyanide p-(trifluoromethoxy)-phenylhydrazone (uncoupler of electron transport chain and ATP synthase), Rotenon+AntimycinA mix (Complex I and III inhibitors)/KCN (cytochrome oxidase inhibitor) or in combination. Seahorse plates were coated with poly-L-lysine or Cell-Tak before seeding 5.10^5^ cells. Respirometric analysis and media acidification analysis using a Seahorse analyzer were performed in RPMI-based media (RPMI, 1mM HEPES, 5mM glucose, pH 7.4).

### 2.4 ATP determination

Cytosolic ATP quantification was performed after 6 (early)/24 (late) hrs of T-cell cultivation in CM-CO/PH Fibs media using ATP bioluminescence assay kit HS II (Roche, Basel, Switzerland) according to the manual.

### 2.5 Semiquantification of ROS production

The general ROS quantity was quantified after 24hrs of T-cell cultivation in CM-CO/PH Fibs media at the 10-minute stage using CM-H_2_DCFDA (Molecular Probes, Eugene, Oregon) on a fluorescence spectrophotometer and the direction of time course was quantified.

### 2.6 Transcriptomic analysis of cytokines and T-cell markers

The mRNA of selected cytokines, and housekeeping genes (**Table 1**) was isolated using the RNeasy kit (Qiagen, Limburg, Netherlands) from 5.10^6^ bovine T-cells after 4,6,24 and 48 hrs of cultivation in CM-CO/PH Fibs and 24 hrs cultivation of human T-cells. Total RNA was quantified using a NanoDrop spectrophotometer (TermoFisher, Waltham MA). For reverse transcription, we used 1000 ng of total RNA. RNA was transcribed using a GrandScript cDNA Synthesis KIT from TataaBiocenter. qPCR was performed in a CFX Connect LightCycler (BioRad, Hercules, CA) using EvaGreen Master Mix (Biotium, Fremont, CA). The amount of relative expression was calculated from the crossing points of each run. The calculation was performed according to the Livak method. Expression of selected mRNA markers in T-cells cultured in RPMI was set to 1.

**Table 1:** Primers for selected cytokines and housekeeping gene.

bIL4	FW: CGCTGAACATCCTCACAACG	RV: TGGCTCCTGTAGATACGCCT
bIFNγ	FW: AGCTGATTCAAATTCCGGTGG	RV: TTACGTTGATGCTCTCCGGC
bTGFβ	FW: CTGACCCGCAGAGAGGAAAT	RV: GCCGGAACTGAACCCGTTAAT
bIL10	FW: AAAACAAGAGCAAGGCGGTG	RV: TGCTTCACTTTTGCATCTTCGT
bHPRT	FW: CTGGCTCGAGATGTGATGAA	RV: CAACAGGTCGGCAAAGAACT
hIL4	FW: CGAGTTGACCGTAACAGACAT	RV: CGTCTTTAGCCTTTCCAAGAAG
hIFNγ	FW: AGCTCTGCATCGTTTTGGGT	RV: TCCGCTACATCTGAATGACCT
hTGFβ	FW: CGACTCGCCAGAGTGGTTAT	RV: GCTAAGGCGAAAGCCCTCAA
hIL10	FW: TACGGCGCTGTCATCGATTT	RV: ACTCATGGCTTTGTAGATGCCT
hHPRT	FW: AGCCCTGGCGTCGTGATTAG	RV: TGATGGCCTCCCATCTCCTT

### 2.7 Immunophenotyping analyses

T-cells were isolated as described above (T-cell isolation) and seeded at a concentration of 1.5.10^6^/ml in 3 ml of the selected medium (CM-CO Fibs, CM-PH Fibs) into 6-well plates and cultivated under standard conditions. For analysis of early induction (6 hrs) of followed cytokines, cells were cultured for 2 hrs and treated with Brefeldin A (BD Biosciences, San Jose, CA) for an additional 4 hrs according to the manufacturer’s protocol to inhibit cytokine secretion. For analysis of late induction (17 hrs) of selected cytokines, cells were incubated with Brefeldin A for 5 hrs and an additional 12 hrs. The reason for not choosing 24 hrs for late induction was technical. T-cells were harvested, washed with PBS/EDTA, and pre-stained with the live/dead marker Zombie- NIR (1:100) for 20 minutes at room temperature (RT) in PBS. Specific surface staining was performed with the antibody mixture (**Table 2**) for 30 min at RT in PBS supplemented with 0.5% BSA. Cells were then fixed for 20 min at RT (CytoFix Fixation Buffer, BD Biosciences, San Jose, CA) and then permeabilized for 15 min at RT with the saponin buffer (PBS supplemented with 0.1% saponin, 0.5% bovine serum albumin). Specific intracellular staining (**Table 2**) was performed by adding a specific antibody cocktail for 20 min at RT. Immunophenotyping was assessed using the BD LSRFortessa and LSRII flow cytometers (BD Biosciences, San Jose, CA), and the data were acquired using the FACS Diva software (version 8.0.1, BD Biosciences, San Jose, CA). Debris was excluded by forward and side scatter gating followed by doublet and dead cell exclusion. Cells were gated: CD3+, CD4+ followed by individual cytokine/T-cell markers. Data were analyzed using the FlowJo software (version 10, BD Biosciences, San Jose, CA).

**Table 2:** Used antibodies for immunophenotyping.

Antibody	Clone	Producer	Dilution	Isotype	Staining type	Fluorochrome conjugation
**Mouse anti-bovine** **TGF-β1,2,3**	1D11	RD Systems	5μl/1.10^6^ cells	IgG1	intra	AF700
**Mouse anti-bovine IL4**	CC303	BioRad	1:5	IgG2a	intra	FITC
**Mouse anti-bovine IFNγ**	CC302	BioRad	1:50	IgG1	intra	AF647
**Mouse anti-bovine IL10**	CC320	Thermo Fisher	1:100	IgG1	intra	PerCP
**Mouse anti-bovine FOXP3**	FOX5A	BioRad	1:100	IgG1	intra	
**Mouse anti-bovine CD25**	IL-A111	Thermo Fisher	1:5	IgG1	surface	PE.
**Mouse anti-bovine CD4**	CC8	BioRad	1:5	IgG2	surface	RPE
**Mouse anti-bovine WC1- γδ T-cells**	ILA29	KingFisher Biotech	1:100	IgG1	surface	n/a
**Mouse anti-bovine CD3**	MM1A	BioRad	1:10	IgG1	surface	n/a
**Mouse anti-bovine TCR1-N24**	GB21A	Novus	1:100	IgG2b	surface	n/a
**Donkey anti-mouse IgG**	n/a	Thermo Fisher	2drops/ml	IgG	n/a	AF488
**Donkey anti-mouse IgG**	n/a	Thermo Fisher	1:1000	IgG	n/a	AF647
**Rat anti-mouse IgG1**	n/a	Thermo Fisher	0.5μg/test	IgG1	n/a	FITC
**Goat anti-mouse IgG**	n/a	Thermo Fisher	1:1000	IgG	n/a	AF633
**Goat anti-mouse IgG2a**	n/a	Thermo Fisher	1:1000	IgG2a	n/a	AF647
**Goat anti-mouse IgG1**	n/a	Thermo Fisher	1:1000	IgG1	n/a	AF647
**Mouse anti-human TGF-β1**	TW4-9E7	BD	1:100	IgG1	intra	PE-CD594
**Mouse anti-human IL4**	8D4-8	BD	1:100	IgG1	intra	BV421
**Mouse anti-human IFNγ**	B27	BD	1:100	IgG1	intra	AF488
**Rat anti-human IL10**	JES3-19F1	BD	1:100	IgG2a	intra	APC
**Mouse anti-human FOXP3**	259D/C7	BD	1:50	IgG1	intra	PE
**Mouse anti-human CD25**	M-A251	BD	1:300	IgG1	surface	BV650
**Mouse anti-human CD4**	SK3	BD	1:300	IgG1	surface	BV510
**Mouse anti-human TCR γδ**	B1	BD	1:150	IgG1	surface	PerCP
**Mouse anti-bovine CD3**	UCHT1	BD	1:300	IgG1	surface	BUV496
**Mouse anti-human CD19**	SJ25C1	BD	1:300	IgG1	surface	BUV395
**Mouse anti-human CD56**	NCAM16.2	BD	1:300	IgG1	surface	BUV395

### 2.8 T-cell proliferation assay

Total T-cells or isolated CD4+ T-cells were stained with Cell Trace Violet (Thermo Fisher, Waltham MA) according to the manufacturer’s instructions. The stained cells were cultured under treatment for 24 hrs. Before analysis, cells were pre-stained with the live/dead marker Zombie NIR as described above. Proliferation of T-cells was measured using the BD LSRII flow cytometer (BD Biosciences, San Jose, CA) as described above. Data were analyzed using ModFit software (version 3.0, Verity Software house, Topsham, ME) and after normalization proliferation index was calculated.

### 2.9 Immunochemical semi-quantification

An equal amount of protein extracts was separated using SDS-polyacrylamide gels and transferred to a PVDF membrane as described previously (http://www.assay-protocol.com). Detection was performed with the appropriate primary antibodies, i.e. phosphorylated PDH, PDH, actin (all from Abcam, Cambridge, UK), and then with secondary antibodies (horseradish peroxidase conjugate and ECL plus blotting kit, GE Healthcare Bio-Sciences Corp, Piscataway, NJ). Quantitative analysis was performed using Image J software densitometry (Rasband,W.S., NIH, US).

### 2.10 Single cell RNAseq analysis of calf pulmonary arteries

Fresh calf distal pulmonary arteries (DPAs) were dissected from the right caudal lung lobe of 3 control and 3 PH calves (14-days hypoxia exposure as described previously (27)). They were subjected to single-cell generation, from which 10,000 cells from each animal were submitted for single-cell RNA seq at a sequencing density of 100,000 reads per cell (Genomic Core at CU Cancer Center, Denver, USA). After extensive bioinformatic based quality controls, the sequencing data of 20,912 cells from control DPAs and 15,692 cells from PH DPAs were included in further studies. The main cell types (Based on Bioconductor R package “SingleR” and “cellDex”) in both control and PH DPAs included smooth muscle cells, fibroblasts, endothelial cells, and macrophages while minor cell types included monocytes, T-cells, NK cells, and neutrophils. We focused on the T-cell population in this study. T-cells were defined by expression of pan T cell markers including CD3D, CD3E, CD3G, ZAP70, CD27 and TRAT1. For T-cell subclusters, samples were normalized using the SCTransform function, followed by Principal Component (PC) level selection. The selected level of PC was used to find cell clusters using the SingleR R package (29). The single-cell RNA sequencing data have been submitted to the Gene Expression Omnibus (GEO) repository at the National Center for Biotechnology Information (NCBI). The dataset can be accessed using the accession number GSE234156. To explore the data further, please visit the GEO website at http://www.ncbi.nlm.nih.gov/geo/.

### 2.11 Statistical analysis

All experiments were performed at least in duplicates and repeated at least three times. ANOVA with Tukey or Sidak tests on the pre-validated data through a normality test or Student´s T-tests (two samples), and Pearson correlation were used for statistical analyses with GraphPad Prism (San Diego, CA). Differences were accepted as statistically significant for p<0.05 (*), p<0.01 (**), p<0.001 (***), p<0.0001 (****).

### 2.12 Data and resource availability

The datasets generated and analyzed during the current study are available from the corresponding author upon reasonable request. No applicable sources were created or analyzed during the current study.

## 3 Results

### 3.1 Single-cell RNA expression analysis of T-cells in intact bovine distal pulmonary arteries of PH calves reveals proinflammatory gene expression

**Figure 1:**
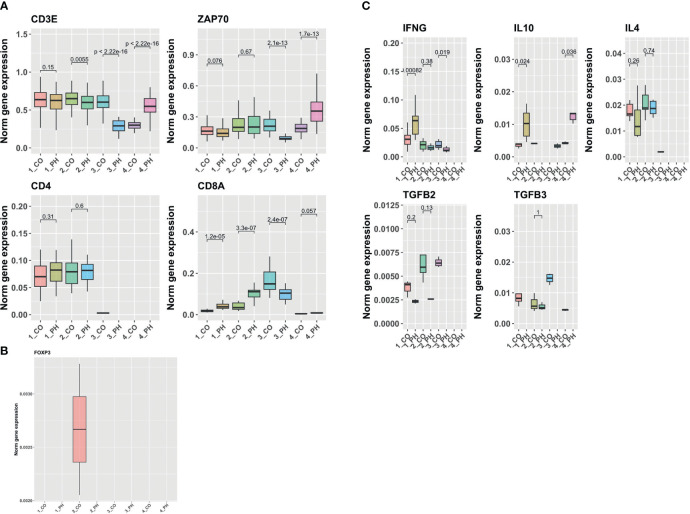
Single-cell RNA sequencing analysis of *in vivo* calf T-cells derived from pulmonary arteries of control/PH calves. **(A)** Analysis of mRNA levels of T cell pan (CD3E and ZAP70) and specific (CD4 for helper T-cells and CD8 for cytotoxic T-cells) marker genes in the T cell subclusters from Control (CO) and PH Distal Pulmonary Arteries (DPAs). **(B)** Analysis of the mRNA levels of regulatory T-cells or Treg marker gene (FOXP3) in the T cell subclusters in CO and PH DPAs. **(C)** Analysis of mRNA levels of cytokines in the T cell subclusters in CO and PH DPAs. Notice that if there is zero value in one of the comparison groups (CO or PH), no statistics can be computed.

We examined gene expression in T-cell populations in distal pulmonary arteries (dPAs) freshly isolated from young calves with or without severe pulmonary hypertension using single-cell RNA sequencing. T-cells represent minority cell type in the walls of dPAs and are in close contact with fibroblasts mainly in the adventitia. We selected T-cell subpopulations expressing general T-cell markers such as CD3E and ZAP70 and T-cell helper marker, CD4+, and marker of cytotoxic T-cells, CD8+ (**Figure 1**). We quantified their expression in T-cells derived from dPAs of CO/PH calves. While general T-cell markers were expressed in all T-cell subclusters presented, the CD4+ marker representing T-helper cells was mainly expressed in the first two subclusters in dPAs from CO/PH calves, whereas subcluster 3 was enriched in CD8+ effector T-cells (**Figure 1A**). Quantification of FOXP3 expression representing Tregs showed its expression in subcluster 2 only, consistent with positivity for CD4 (**Figure 1B**). Interestingly, FOXP3 mRNA expression was detectable only in T-cells from dPAs of control calves (CO), whereas no signal was observed in dPAs from PH calves. Furthermore, we quantified the mRNA expression of previously selected markers representing Th1 cellular immunity (IFNγ), Th2 humoral immunity (IL4), and Tregs suppression tolerance immunity (IL10, TGFβ) of T-cell subclusters from dPAs of CO/PH calves. We observed increased expression of IFNγ in subcluster 1 of CD4+ T-cells from dPAs of PH calves, increase or decrease in IL10 in subclusters 1 and 2 of CD4+ T-cells from dPAs of PH calves, whereas a decrease of IL4 in subclusters 1 and 2 of CD4+ T-cells from dPAs of PH calves (**Figure 1C**). The expression of TGFβ2/3 was decreased in subclusters 1 and 2 of CD4+ T-cells from dPAs of PH calves (**Figure 1C**).

### 3.2 Conditioned media from bovine PH- and human IPAH- fibroblasts controls CD4+ T-cell polarization

**Figure 2:**
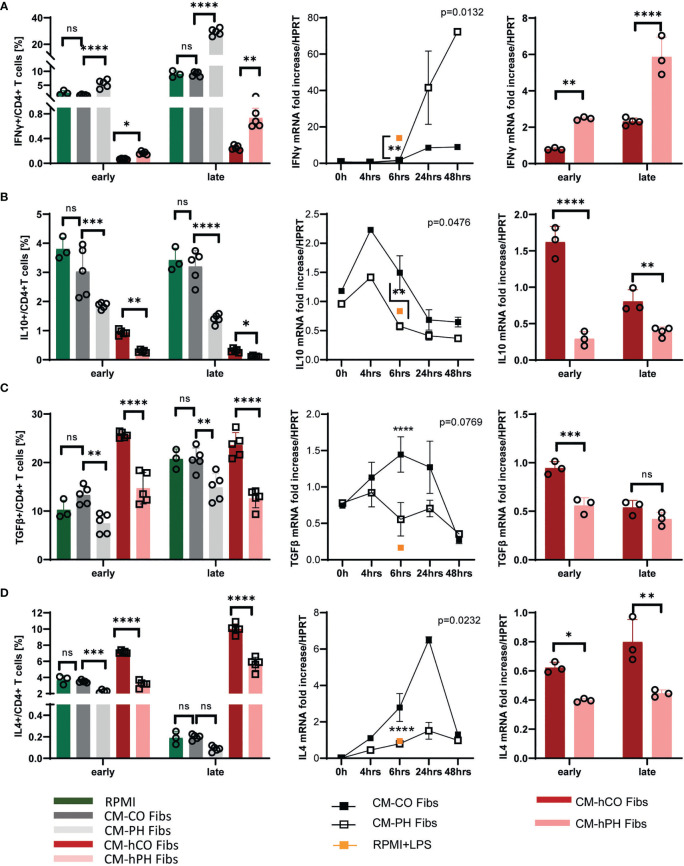
Analysis of cytokines and markers and their mRNA expression profile of bovine/human T-cells exposed to CM-(h)CO/PH Fibs. **(A)** Frequency of IFNγ positive bovine/human T-cells per CD4+ T-cells (left panel) and mRNA expression of IFNγ in the period of 0-48 hrs (bovine, middle panel) and early and late time period (human, right panel) in bovine/human T-cells exposed to CM-(h)CO/PH Fibs and RPMI as a control media for early/late time period, n=3-5; Pearson correlation p=0.0132, n=3-5. **(B)** Frequency of IL10-positive bovine/human T-cells per CD4+ T-cells (left panel) and mRNA expression of IL10 in the period of 0-48 hrs (bovine, middle panel) and early and late time period (human, right panel) in bovine/human T-cells exposed to CM-(h)CO/PH Fibs and RPMI as a control media for early/late time period, n=3-5; Pearson correlation p=0.0476, n=3-5. **(C)** Frequency of TGFβ- positive bovine/human T-cells per CD4+ T-cells (left panel) and mRNA expression of TGFβ in the period of 0-48 hrs (bovine, middle panel) and early and late time period (human, right panel) in bovine/human T-cells exposed to CM-(h)CO/PH Fibs and RPMI as a control media for early/late time period, n=3-5; Pearson correlation p=0.0769, n=3-5. **(D)** Frequency of IL4 positive bovine/human T-cells per CD4+ T-cells (left panel) and mRNA expression of IL4 in the period of 0-48 hrs (bovine, middle panel) and early and late time period (human, right panel) in bovine/human T-cells exposed to CM-(h)CO/PH Fibs and RPMI as a control media for early/late time period, n=3-5; Pearson correlation p=0.0232, n=3-5. In mRNA expression experiments with bovine T-cells proinflammatory control was performed by LPS/nigericin treatment for 6 hrs, n=3 (orange square). p<0.05 (*), p<0.01 (**), p<0.001 (***), p<0.0001 (****), not significant (ns).

We have previously shown that macrophage activation in the adventitia of hypertensive vessels is triggered by factors released by fibroblasts of the tunica externa (12, 18). Here, we immunophenotyped the entire CD4+ T-cell population exposed to conditioned media from control and PH/IPAH fibroblasts (CM -(h) CO/(h) PH Fibs) using a flow cytometry approach. Because of the lack of bovine-specific antibodies on the market and the limited number of human samples, we focused on the analysis of cytokine production reflecting T-cell type and mRNA expression at two exposure time points, representing the early (6hrs) and late (17/24 hrs) cytokine expression (**Figure 2**). We observed that CD4+ T-cells from both bovine and humans exposed to conditioned media of IPAH/PH fibroblasts (CM -(h) PH Fibs) showed higher positivity of IFNγ whereas the positivity of IL10, IL4 and TGFβ was decreased compared with CD4+ T-cells in conditioned media of control fibroblasts (**Figures 2A–D**, left panel). In general, the abundance of cells positive for individual cytokines corresponded to their RNA expression at early and late time points of exposure of bovine and human T-cells to conditioned media (**Figure 2**, left vs. middle and right panels).

Bovine and human CD4+ T-cells cultured in CM -(h) PH Fibs showed a significantly increased IFNγ Th1 response, indicating the T-cell differentiation to an inflammatory state (**Figure 2A**). IFNγ produced by bovine and human CD4+ T-cells in CM -(h) PH Fibs closely matched its mRNA expression, and specifically their late expression showed a significant increase in IFNγ positive T-cells at the late time point (**Figure 2A**).

The amount of IL10 expressed relatively early (peak at 4 hrs) was decreased in bovine and human T-cells after exposure to CM -(h) PH Fibs compared with CM -(h) CO Fibs cultivation (**Figure 2B**). Similarly, TGFβ positivity significantly decreased in bovine and human T-cells when cultured in CM-PH Fibs at both time points, whereas their gene expression was relatively stable at the time points examined (**Figure 2C**).

IL4, which has mainly anti-inflammatory properties, was significantly decreased in bovine T-cells in CM-PH Fibs media only at an early time point of cultivation, whereas IL4 signal in human T-cells decreased significantly at early and late time points after CM -hPH Fibs exposure (**Figure 2D**). This corresponded with high positivity of IFNγ Th1 response in these cells. The low IL4 positivity of bovine T-cells (less than 1%) cultured in CM-CO/PH Fibs corresponded to low mRNA expression at the late time point of cultivation. (**Figure 2D**). Note that time point 0 of T-cell exposure corresponds to RPMI media.

### 3.3 Tregs are downregulated in T-cells cultivated in conditioned media of PH- and IPAH fibroblasts

**Figure 3:**
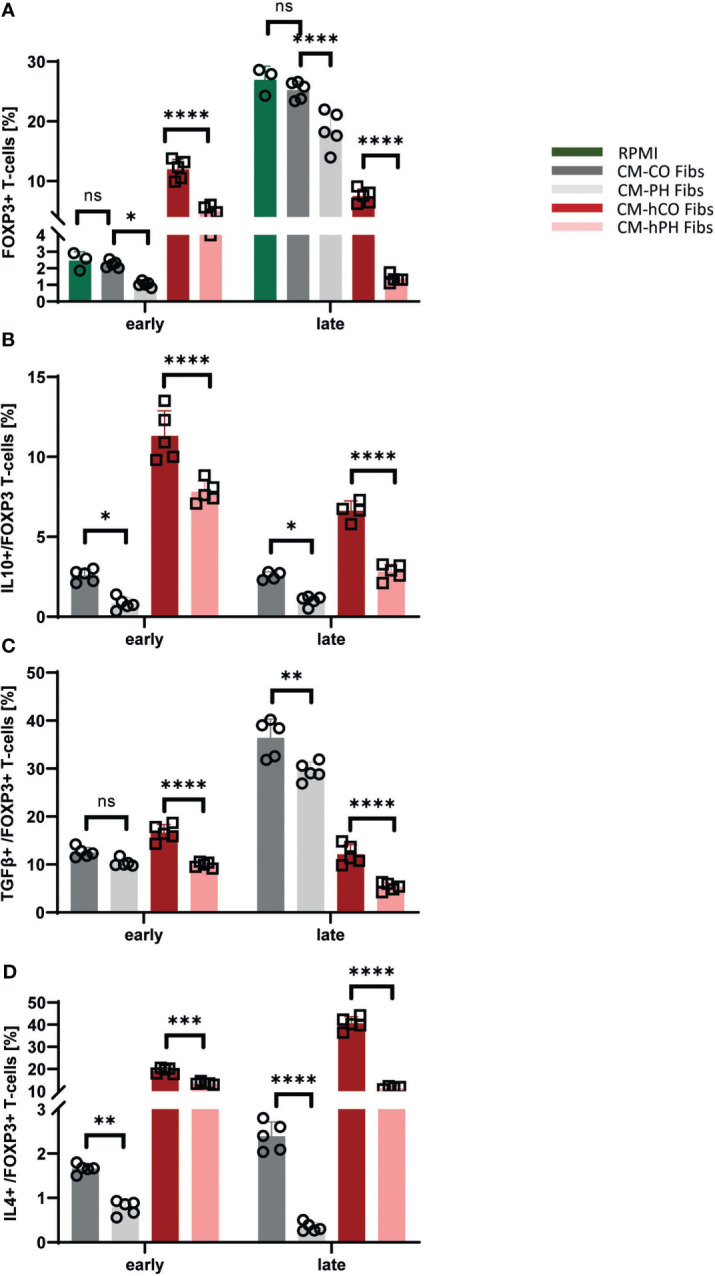
Analysis of cytokines and T-cell markers within the FOXP3 human/bovine T-cells exposed to CM-(h)CO/PH Fibs. **(A)** Frequency of FOXP3 cells per bovine/human CD4+ T-cells exposed to CM-(h)CO/PH Fibs and RPMI as a control media for early/late time point, n=3-5. **(B)** Frequency of IL10-positive cells per FOXP3 bovine/human T-cell population exposed to CM-(h)CO/PH Fibs for early/late time point, n=3-5. **(C)** Frequency of TGFβ-positive cells per FOXP3 bovine/human T-cell population exposed to CM-(h)CO/PH Fibs for early/late time point, n=3-5. **(D)** Frequency of IL4-positive cells per FOXP3 bovine/human T-cell population exposed to CM-(h)CO/PH Fibs for early/late time point, n=3-5. p<0.05 (*), p<0.01 (**), p<0.001 (***), p<0.0001 (****), not significant (ns).

The decreased IL10 and TGFβ positivity in bovine and human CD4+ T-cells cultured in CM -(h) PH Fibs suggested a decreased presence of Tregs compared with CM -(h) CO Fibs (**Figures 2B, C**). Indeed, the population of FOXP3 T-cell subset within bovine/human entire CD4+ T-cell populations was significantly decreased at both time points in CM-(h) PH Fibs (**Figure 3A**). The decreased number of bovine FOXP3 T-cells exposed to CM-PH Fibs corresponded with the undetected mRNA expression of FOXP3 in *in situ* samples from hypertensive dPAs (**Figures 3A**, **1B**). The decrease was even more pronounced in FOXP3 population of human T-cells during late exposure to CM -hPH Fibs (**Figure 3A**). As a subtype of entire CD4+ T-cells all FOXP3 T-cells were CD4+. Interestingly, the abundance of the cytokines IL10, TGFβ, and IL4 in the FOXP3 subset of bovine T-cells were highly consistent with the abundances observed in the CD4+ T subset when cultured in CM-PH Fibs vs. CM-CO Fibs (**Figures 3B–D** vs. **Figures 2B–D**). Only the cytokine TGFβ was present at slightly higher levels in the FOXP3 subset of bovine T-cells when cultured in CM-CO/PH Fibs, especially at the late time point compared with the entire CD4+ T-cell population (**Figure 3C** vs. **Figure 2C**). The cytokine IL4 was weakly expressed in bovine CD4+ T-cells at the late time point, but higher IL4 positivity was observed in the subpopulation of bovine FOXP3 T-cells cultured in CM-CO Fibs at this late time point. (**Figure 3D** vs. **2D**). Interestingly, the number of IL10+, TGFβ+, and IL4+ human T-cells was significantly higher in the FOXP3 subset of human T-cells compared with entire human CD4+ T-cells at all cultivation conditions and time points (**Figures 3B–D** vs. **2B–D**).

### 3.4 Pro-inflammatory bovine T-cells induce glycolytic metabolism and proliferation and create a prooxidant intracellular milieu upon exposure to CM-PH Fibs

**Figure 4:**
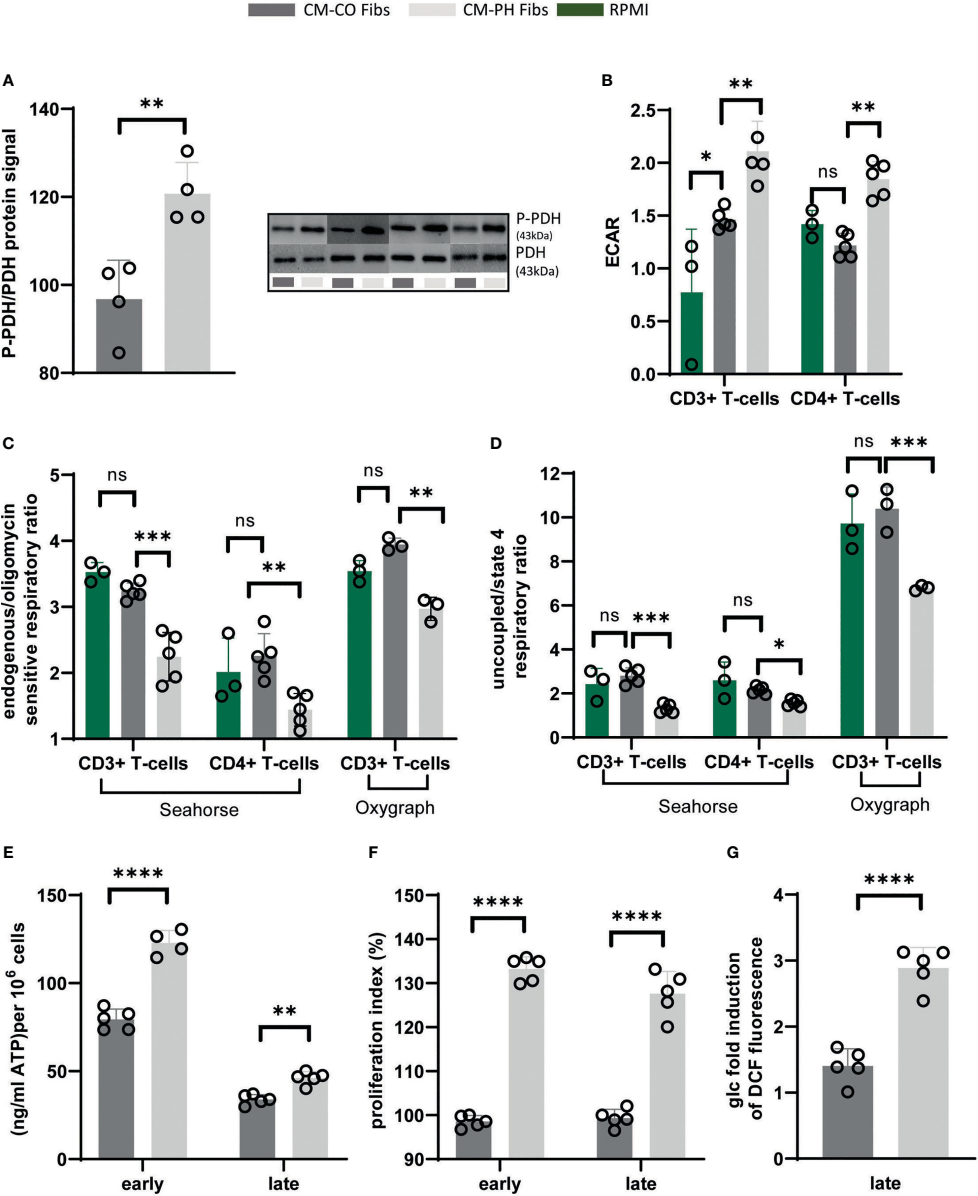
Energy metabolism, redox status and proliferation of bovine T-cells exposed to CM-CO/PH Fibs. **(A)** P-PDH/PDH protein ratio of CD4+ T-cells in CM-CO/PH Fibs after 24-hour cultivation, n=4. MW of (P)PDH signal is 43kDa. **(B)** Lactate production expressed as media acidification (ECAR) of total (CD3+)/CD4+ T-cells in CM-CO/PH Fibs after 24-hour cultivation, n=3-5. **(C)** Oxidative phosphorylation activity expressed as endogenous/oligomycin-sensitive respiratory ratio of total (CD3+)/CD4+ T-cells in CM-CO/PH Fibs after 24-hour cultivation, n=3-5. **(D)** Maximum mitochondrial respiration analysis expressed as uncoupled/oligomycin-sensitive respiration ratio of total (CD3+)/CD4+ T-cells in CM-CO/PH Fibs after 24-hour cultivation, n=3-5. **(E)** Quantification of cytosolic ATP of T-cells in CM-CO/PH Fibs at early/late point of cultivation, n=4-5. **(F)** Proliferation of T-cells in CM-CO/PH Fibs at early/late point of cultivation, n=5. **(G)** Cytosolic redox status expressed as glucose-induced increase in DCF fluorescence in T-cells in CM-CO/PH Fibs after 24-hour cultivation, n=5. p<0.05 (*), p<0.01 (**), p<0.001 (***), p<0.0001 (****), not significant (ns).

To understand how the pro-inflammatory polarization of bovine T-cells exposed to CM-PH Fibs correlates with the metabolic status of these cells, we performed an analysis of energy and redox metabolism. Analysis of the energy metabolism of T-cells at the late time point of exposure to CM-CO/PH Fibs reveals a switch to glycolytic metabolism in T-cells cultured in CM-PH Fibs compared with CM-CO Fibs media (**Figures 4A-D**). Thus, we observed increased phosphorylation of pyruvate dehydrogenase (PDH) in bovine T-cells, which was detected immunochemically and expressed as an increase in the P-PDH/PDH ratio of approximately 20% (**Figure 4A**). The increased phosphorylation of PDH results in pyruvate being directed to lactate production rather than mitochondrial breakdown in T-cells cultured in CM-PH Fibs. The increased lactate production was determined as the acidification of the medium (extracellular acid production/ECAR), which increased by 42% when T-cells were cultured in CM-PH Fibs (**Figure 4B**). The metabolic switch was also documented by the respiratory control ratio (endogenous/oligomycin-sensitive respiratory ratio), which determines the mitochondrial activity of oxidative phosphorylation/ATP synthesis in the context of endogenous respiration, which decreased by approximately 30% in T-cells cultured in CM-PH Fibs (**Figure 4C**). This corresponded with a greater than 50% decrease in maximal mitochondrial respiratory capacity in T-cells cultured for 24 hrs in CM-PH Fibs (**Figure 4D**). Interestingly, glucose induction of endogenous respiration was more pronounced in T-cells cultured in CM-CO Fibs (**Figure S1A**). We used T-cells treated with LPS/nigericin as control cells that exhibited increased Warburg metabolism because LPS reduces oxygen consumption *via* TLR4 signaling (29, 30), which correlated with data obtained for T-cells in CM-PH Fibs (**Figure S1B**). Activation of glycolytic metabolism of T-cells in CM-PH Fibs media is based on induction of cytokines/metabolites derived from fibroblasts and therefore does not require cell-to-cell contact. Most of the respiration data were obtained by analyzing oxygen consumption with the Seahorse analyzer, which requires fewer cells per analysis. However, the data were confirmed by analysis with the Oxygraph analyzer, the gold standard of respiratory analysis (**Figures 4C, D**, **S1A, B**). In parallel with the respiratory analyzes performed on the entire population of bovine T-cells, both CD4+ and CD8+ T-cells, we also isolated CD4+ T-cells for analysis. This confirmed the respirometry data obtained with the entire T-cell population (**Figures 4C, D**, **S1A**, S1B). To further investigate the energy metabolism of T-cells, we determined the intracellular ATP content after their cultivation in CM-CO/PH Fibs. We detected an increase in intracellular ATP of T-cells cultured in CM-PH Fibs depending on the duration of cultivation, i.e., 3, 6, 17, and 24 hrs (early (6 hrs) and late (24 hrs) exposure results are in **Figure 4E**). This increase was comparable to that observed in T-cells treated with LPS/nigericin (**Figure S1C**). The glycolytic switch induced proliferation of T-cells in CM-PH Fibs through whole studying period compared with CM-CO Fibs (**Figure 4F**).

The decrease in mitochondrial oxidative phosphorylation is often associated with changes in intracellular redox status. Therefore, we determined the overall intracellular redox status. T-cells cultured in CM-PH Fibs showed increased production of reactive oxygen species (ROS) after 24 hrs of incubation, i.e., an increase of approximately 32%, similar to the ROS increase after LPS treatment of T-cells in RPMI media, which also exhibit a glycolytic shift (**Figures 4G**, **S1D**).

### 3.5 γδ+ T-cells play a role in cytokine production in bovine PH-associated environment of adventitia

Although the existence of Tregs has been demonstrated in bovine blood, it has recently been discovered that 30-60% of the peripheral blood mononuclear cells of young sheep and cattle represent γδ+ T-cells (30). These are CD4- CD8- T-cells that express a different T-cell receptor termed γδ TCR, compared with classical CD4+ CD8+ T-cells. In addition, a large proportion of these T-cells in cattle express the unique costimulatory molecule WC1. It has been reported that the subpopulation of WC1+ γδ+ T-cells in cattle is more likely to function as immunoregulatory cells ex vivo than CD4+ CD25+high FOXP3 T-cells (17).

**Figure 5:**
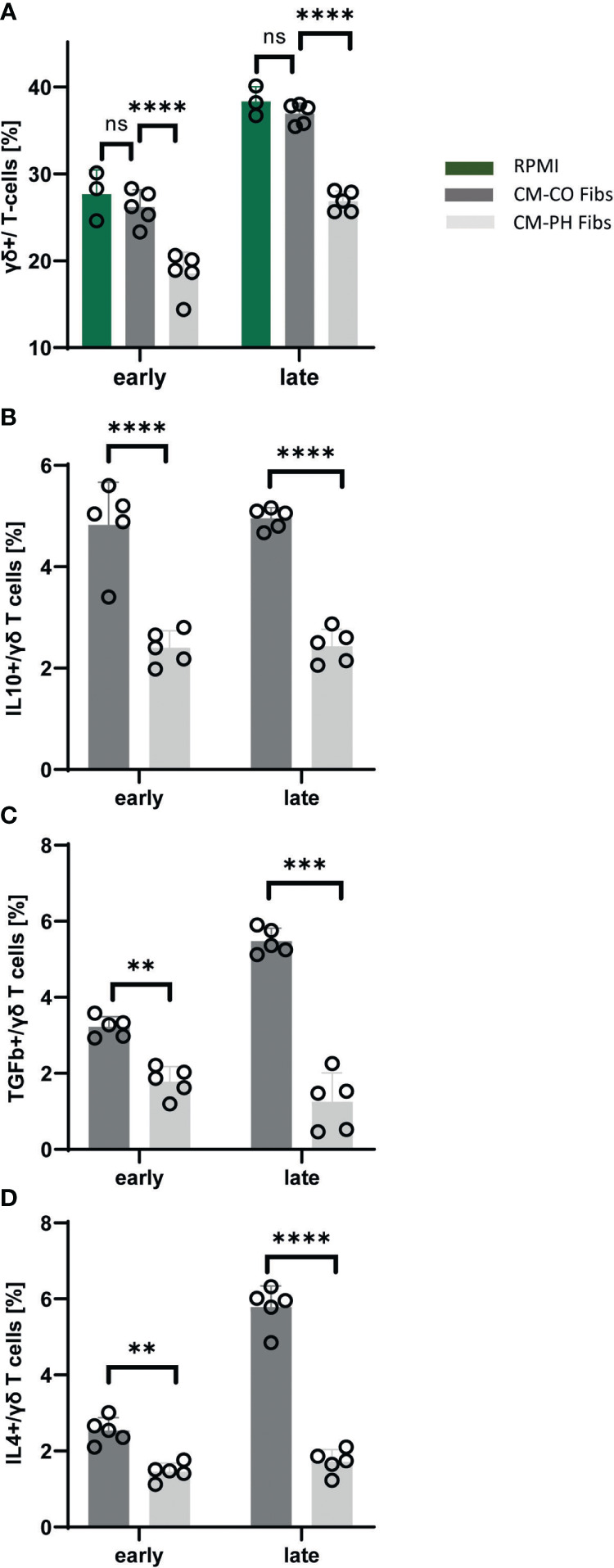
Analysis of cytokines and T-cell markers within bovine γδ+T-cell population exposed to CM-CO/PH Fibs. **(A)** Frequency of γδ+ cells per bovine T-cell population exposed to CM-CO/PH Fibs and RPMI as a control media at early/late point of cultivation, n=5. **(B)** Frequency of IL10-positive cells per γδ+ bovine T-cell population exposed to CM-CO/PH Fibs at early/late point of cultivation, n=5. **(C)** Frequency of TGFβ-positive cells per γδ+ bovine T-cell population exposed to CM-CO/PH Fibs at early/late point of cultivation, n=5. **(D)** Frequency of IL4-positive cells per γδ+ bovine T-cell population exposed to CM-CO/PH Fibs at early/late point of cultivation, n=5. p<0.01 (**), p<0.001 (***), p<0.0001 (****), not significant (ns).

Quantification of the abundance of bovine γδ+ T-cells showed a significant decrease when cultured in CM-PH Fibs compared with CM-CO Fibs at both time points (**Figure 5A**). Note that the number of γδ+ human T-cells in all CM cultures was limited (below 0.15%). γδ+ T-cells were CD4- and did not express IFNγ (not shown). We were interested in the cytokines γδ+ T-cells produce. Because they have regulatory properties, we focused on the presence of IL10, TGFβ, and IL4 cytokines in this T-cell subset. Compared with the CD4+ subset and also with the FOXP3 subset, we observed a significantly lower frequency of TGFβ+ cells under all cultivation conditions and at all-time points (**Figures 2C**, **3C**, **5C**). On the other hand, γδ+ T-cells cultured in CM-CO Fibs significantly increased the frequency of IL4+ cells at the late time point compared with CD4+ T-cells (**Figures 5D**, **2A**) and also slightly, but not significantly, increased IL10+ cells at both time points compared with CD4+ T-cells (**Figures 5B**, **2B**). All cytokines examined were less abundant in γδ+ T-cells cultured in CM-PH Fibs than in CM-CO Fibs, suggesting their weaker suppressive phenotype (**Figures 5B–D**).

### 3.6 SAHA, the HDAC class I inhibitor, suppresses pro-inflammatory polarization and glycolytic metabolism of T-cells induced by CM-PH Fibs cultivation

We wanted to determine whether the metabolic reprogramming of PH Fibs that triggers their proliferation and pro-inflammation has direct effects on T-cell differentiation. We have previously shown that HDAC class I inhibitors can reverse the metabolic abnormalities observed in PH Fibs and thereby reduce hypoxia-induced PH in calves (16), in part *via* the miR-124/PTBP1/PKM pathway (31). Therefore, we sought to determine whether restoring normal fibroblast metabolism using HDAC inhibitor SAHA would inhibit the polarization of T-cells toward proinflammatory signaling. We treated bovine CO and PH Fibs cells with SAHA for 72 hrs and used the resulting media (CM-CO/PH Fibs+SAHA) to culture bovine T-cells for a maximum of 24 hrs. Unless otherwise indicated, we performed analyzes at late (metabolic analysis) or early/late (expression analysis) time points.

**Figure 6:**
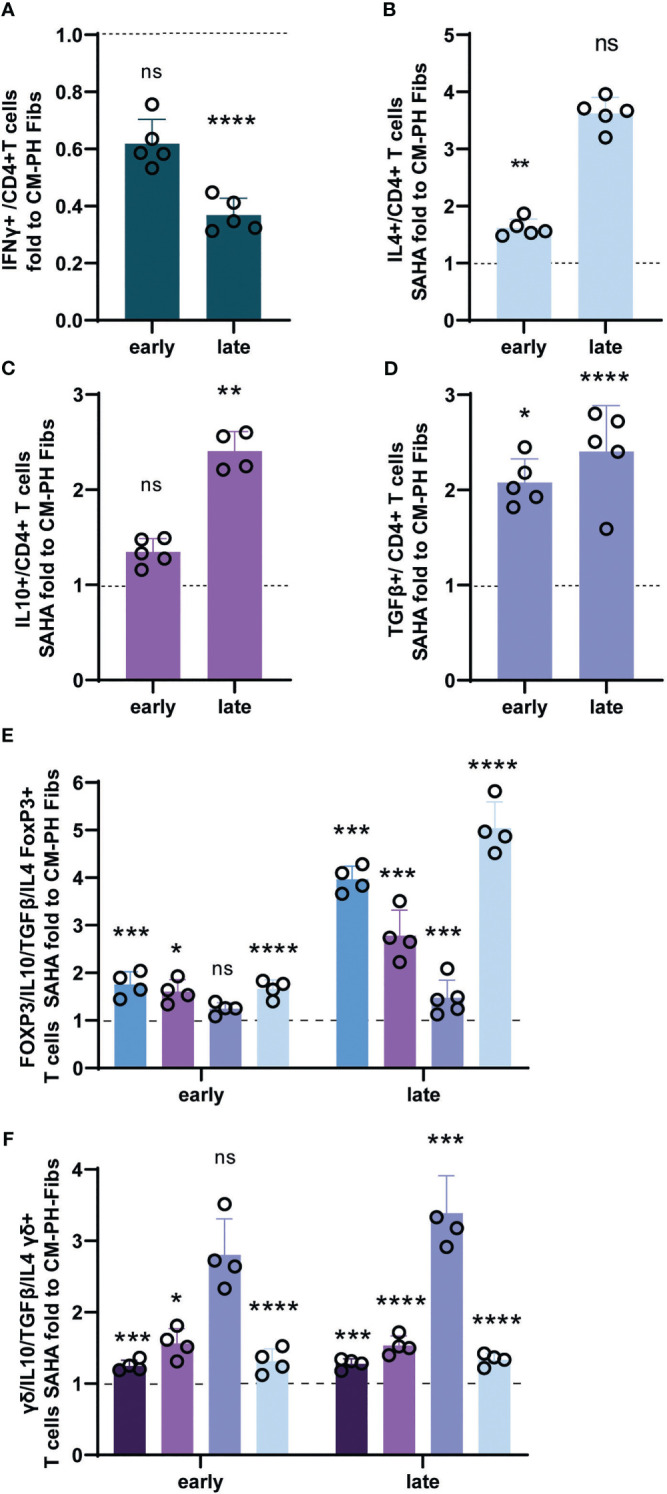
Analysis of cytokines and markers of bovine T-cells exposed to CM-CO/PH Fibs+SAHA. **(A)** Fold change of frequency of IFNγ -positive bovine CD4+ T-cells exposed to CM-CO/PH Fibs+SAHA for early/late time period to CM-CO/PH Fibs, n=5. **(B)** Fold change of frequency of IL4-positive bovine CD4+ T-cells exposed to CM-CO/PH Fibs+SAHA for early/late time period to CM-CO/PH Fibs, n=5. **(C)** Fold change of frequency of IL10-positive bovine CD4+ T-cells exposed to CM-CO/PH Fibs+SAHA for early/late time period to CM-CO/PH Fibs, n=5. **(D)** Fold change of frequency of TGFβ -positive bovine CD4+ T-cells exposed to CM-CO/PH Fibs+SAHA for early/late time period to CM-CO/PH Fibs, n=5. **(E)** Fold change of frequency of FOXP3 T-cells and IL10+/TGFβ+/IL4+ cells per FOXP3 exposed to CM-CO/PH Fibs+SAHA for early/late time point to CM-CO/PH Fibs, n=4-5. **(F)** Fold change of frequency of γδ+ T-cells and IL10+/TGFβ+/IL4+ cells per γδ+ exposed to CM-CO/PH Fibs+SAHA for early/late time point to CM-CO/PH Fibs, n=3-5. p<0.05 (*), p<0.01 (**), p<0.001 (***), p<0.0001 (****), not significant (ns).

We found that CM-PH Fibs+SAHA significantly suppressed proinflammatory T-cell differentiation, as documented by a decreased amount of IFNγ-producing CD4+ T- cells, and increased the number of CD4+ T- cells positive for IL4, IL10, and TGFβ at both time points, reaching levels comparable to those of CM-CO Fibs (**Figures 6A–D**). The decrease in IFNγ+ T- cells at the late time point corresponded to the downregulation of mRNA expression (**Figures 6A**, **S2A**). The increase in the number of Th2-producing IL4+ T- cells after CM-PH Fibs + SAHA culturing was small but significant, especially at the early time point, and corresponded to overall weak expression, especially at the late time point of incubation (**Figures 6B**, **S2B**). The recovery of IL10+ and TGFβ+ T- cell abundance corresponded to their upregulation of expression after both time points of exposure to CM-PH Fibs+SAHA compared with CM-PH Fibs, indicating an increase in Treg cell abundance (**Figures 6C, D**, **S2C, D**). When bovine T- cells were cultured in CM-PH Fibs+SAHA, the number of FOXP3 T-cells fully recovered at both time points (**Figure 6E**). In addition, culturing FOXP3 T- cells in CM-PH Fibs+SAHA resulted in a complete recovery of the levels of cytokines IL10, TGFβ, and IL4 to those observed when exposed to CM-CO Fibs (**Figure 6E**), supporting the beneficial effect of CM-PH Fibs+SAHA on reducing T-cell inflammation. There was no significant difference between the number of FOXP3 T- cells cultured in CM-CO Fibs+SAHA and CM-CO Fibs, similar to cytokine production under these conditions (not shown). Interestingly, culturing T-cells in CM-PH Fibs+SAHA also restored the number of γδ+ T- cells to the level observed when T-cells were exposed to CM-CO Fibs at both time points (**Figure 6F**). The presence of CM-PH Fib+SAHA also had a recovery effect on the abundance of the cytokines studied (ie, IL4, IL10, TGFβ) in γδ+ T- cells compared with the abundance observed in γδ+ T- cells exposed to CM-PH Fibs (**Figure 6F**). We did not observe a significant effect of CM-CO Fibs+SAHA on the presence of γδ+ T- cells or the frequency of cytokines in the γδ+ T subset compared with CM-CO Fibs (not shown).

**Figure 7:**
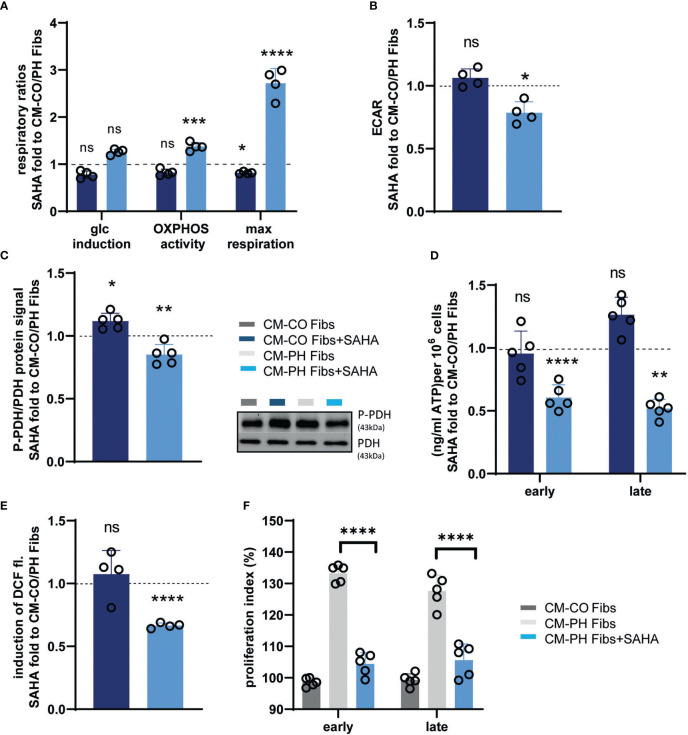
Analysis of metabolism, redox status and proliferation of bovine T-cells cultured in CM-CO/PH Fibs ± SAHA. **(A)** Glucose fold induced endogenous respiration, oxidative phosphorylation activity (OXPHOS activity) and maximal mitochondrial respiratory capacity of T-cells cultured in CM-CO/PH Fibs ± SAHA for 24 hrs expressed as fold change to CM-CO/PH Fibs T-cells, n=4-5. **(B)** Lactate production expressed as media acidification (ECAR) of T-cells in CM-CO/PH Fibs ± SAHA after 24 hrs of cultivation expressed as fold change to CM-CO/PH Fibs T-cells, n=4-5. **(C)** P-PDH/PDH protein ratio of T-cells in CM-CO/PH Fibs ± SAHA after 24 hrs of cultivation expressed as fold change to CM-CO/PH Fibs T-cells, n=4-5. **(D)** Quantification of cytosolic ATP of T-cells in CM-CO/PH Fibs ± SAHA at early/late time point of cultivation expressed as fold change to CM-CO/PH Fibs T-cells, n=5. **(E)** Cytosolic redox status expressed as glucose-induced increase in DCF fluorescence in T-cells in CM-CO/PH Fibs ± SAHA after 24 hrs of cultivation expressed as fold change to CM-CO/PH Fibs T-cells, n=4-5. **(F)** Proliferation of T-cells in CM-CO/PH Fibs +/- SAHA at early/late point of cultivation, n=5. p<0.05 (*), p<0.01 (**), p<0.001 (***), p<0.0001 (****), not significant (ns).

We asked whether the anti-inflammatory effect of CM-PH Fib+SAHA on T-cell differentiation also affects T-cell metabolism. We observed a recovery of mitochondrial oxidative phosphorylation/ATP synthesis, as evidenced by an increased ratio of respiratory control and maximal respiratory capacity of T- cells cultured in CM-PH Fibs + SAHA (**Figure 7A**). No significant change was observed in T- cells treated with CM-CO Fibs + SAHA compared with T- cells in CM-CO Fibs (**Figure 7A**). Restoration of mitochondrial ATP synthase activity in T- cells cultured in CM-PH Fibs+SAHA was supported by a significant decrease in media acidification; however, such an effect was not evident in T- cells cultured in CM-CO Fibs (**Figure 7B**). Culturing in CM-PH Fibs+SAHA also decreased phosphorylation of PDH in T- cells, which correlates with restoration of pyruvate oxidation in mitochondria instead of fermentation to lactate (**Figures 7A–C**). Interestingly, cytosolic ATP content in T- cells decreased after early and late exposure to CM-PH Fibs+SAHA (**Figure 7D**). Switching energy metabolism to mitochondrial oxidative phosphorylation in T- cells cultured in CM-PH Fibs+SAHA also significantly suppressed cytosolic ROS production (**Figure 7E**). The switch of metabolism to oxidative phosphorylation and the decreased intracellular oxidative status of T- cells exposed to CM-PH Fibs+SAHA significantly reduced T- cell proliferation (**Figure 7F**).

## 4 Discussion

We have demonstrated the active role of pulmonary fibroblasts in activation and polarization of human/bovine CD4+ T-cells, demonstrating their crucial role in PH mediated inflammation. This complements previous data showing that pulmonary fibroblasts from PH animals (PH-Fibs) secrete lipid mediators, chemokines, cytokines, and other factors to promote recruitment and activation of monocytes/macrophages, which then infiltrate the adventitia of the PH vascular wall and activate them for inflammation (18). Here, we focused on CD4+ helper T-cells as the key coordinators of the immune response. We demonstrated that CD4+ T-cells present in the distal pulmonary artery of PH calves reprogram their expression toward inflammation, as evidenced by increased expression of IFNγ and decreased expression of IL4, TGFβ and the absence of Tregs marker, FOXP3. *In vitro* experiments exposing isolated bovine/human T-cells to conditioned media of IPAH/PH Fibs (CM-(h)PH Fibs) confirmed the increased inflammatory polarization observed *in situ*. Human/bovine T-cells differentiated toward proinflammatory Th1, whereas polarization toward Th2 and suppressive regulatory Tregs and γδ+ T subset was reduced in CM-(h)PH Fibs. Interestingly, the amount of immunosuppressive cytokines produced by Tregs (FOXP3) was also reduced. Thus, fibroblasts were shown to respond to and produce a variety of inflammatory signals in addition to growth factors that influence immune cell behavior. Similarly, fibroblast-derived IL6, IL8, and IL11 have been shown to activate and attract neutrophils during neutrophil inflammation in asthma but also to activate T-cells by increasing CD40L expression (32, 33).

It has also been suggested that interaction between fibroblasts and immune cells increases the production and degradation of ECM proteins and is thus involved in vascular wall remodeling. Moreover, proinflammatory differentiation of T-cells after CM-(h)PH Fibs exposure correlated with metabolic changes in these cells. We observed increased aerobic glycolysis and lactate production along with induction of prooxidant metabolism, and this metabolic activation resulted in increased proliferation of CD4+ T-cells. Epigenetic changes induced by the HDAC inhibitor SAHA in PH Fibs restored their metabolism and also decreased their proinflammatory potential toward exposed T-cells, as previously shown for monocyte/macrophages (34). Interestingly, cell-to-cell contact was not required for communication between PH Fibs and T-cells. This finding agrees well with the previously observed decreased ability of PH Fibs treated with class I HDAC inhibitors to induce monocyte migration and proinflammatory activation (34), otherwise associated with vascular remodeling. In addition to local inflammation involved in pulmonary vascular wall remodeling, PH patients have elevated serum levels of mostly proinflammatory mediators including IL6, IL8, TNFα, CCL5, CCL7, CCL4, MIF, TNFβ, CXCL9, IL3, and TRAIL, which are associated with reduced survival (35–37). Although many of the above serum chemokines and interleukins affect the recruitment of mononuclear cells to the endothelium or migration and proliferation of pulmonary artery smooth muscle cells (PASMC), the association between altered serum cytokine levels and local pulmonary vascular inflammation is lacking. In any case, remodeling of the pulmonary vasculature is an active process driving disease development, and dysregulated immunity not only in the adventitia appears to be a key component (38).

The imbalance of T-cell subsets has been demonstrated in many pathologies (38–44). The key role of Tregs in limiting and downregulating the immune response makes them particularly important in the prevention of autoimmunity (45). They secrete IL10, thereby preventing antigen presentation and DC maturation. They also inhibit T-cell activation and polarization by expressing granzyme B and CD73. Their metabolic state has been shown to be important for their suppressive function, and its targeting offers new therapeutic opportunities (46). They utilize a variety of metabolic pathways but take up a considerable amount of glucose ex vivo, and their highly glycolytic metabolism is required for their activation and proliferation (47, 48). Their role has also been documented in PH, where depletion of CD4+ T-cells in immunocompetent animals increased susceptibility to PH (20). Similarly, their administration to mice with hypoxia-induced PH improved their phenotype and vascular remodeling by upregulating IL10 and significantly reducing IL1β, IL6, and MCP1 (49). Indeed, we observed a significant reduction in the FOXP3 T-cell subset over the entire period of exposure of human/bovine T-cells to CM-(h)PH Fibs corresponding to undetectable FOXP3 expression in distal pulmonary arteries of calves with severe PH using the scRNA sequencing approach. Moreover, these FOXP3 cells exhibited reduced numbers of IL10- and TGFβ positive cells. Their abundance was significantly restored by inhibition of metabolic reprogramming of PH Fibs by class I HDAC, supporting metabolically controlled secretion of mediators by PH Fibs (34). Apart from positive regulation of Tregs, imbalanced signaling through TGF-β contributes greatly to dysregulated vascular cell proliferation in pulmonary hypertension and affects the majority of cell types in the pulmonary artery wall layers. Its complex signaling and distinct temporal and cell-specific expression patterns highlight its role in vascular remodeling in PH. Further studies are needed to clarify its character within PH development.

Another T-cell subset capable of regulating T immune cells in calves are non-conventional γδ T-cells, which are known as innate-like cells and can utilize T-cell receptor (TCR) as well as other types of receptor systems such as pattern recognition receptors (PRR) and natural killer receptors (NKR). They are further classified based on the presence of the co-receptor WC and WC1+ and can be further subdivided into subpopulations depending on which WC1 genes of the multigenic array they express (reviewed in [30)]. Young ruminants have a significantly higher representation of γδ+ T-cells compared to mice or humans. Unlike conventional α,β TCR containing T-cells, they do not require MHC presentation and therefore can respond to cytokines alone in cattle. Ruminant γδ+ T-cells are recognized as regulatory cells producing IL10 and TGFβ and are considered the major Treg population of cattle rather than FOXP3 T-cells compared to humans and mice (17, 50, 51). We recognized both regulatory T-cell populations, i.e. Tregs and γδ+ T subset, in our calf model, and both were downregulated when cultured in CM-PH Fibs. Note, that number of γδ+ T-cells was insignificant in human T-cells exposed to CM-hPH Fibs. Bovine γδ+ T-cells exposed to CM-PH Fibs showed significantly decreased numbers of TGFβ, IL4, and IL10 positivity. However, cytokine markers as well as the number of both regulatory T subsets recovered when exposed to CM-PH Fibs+SAHA, suggesting that stimuli released from metabolically restored PH fibroblasts may regulate the local immune response.

Clearly, other immune cells besides T-cells will be involved in remodeling of the arterial wall as PH develops. We have recently shown that the pro-inflammatory and pro-remodeling phenotype of macrophages and the activation of the complement cascade, especially its alternative pathway, is a key mechanism that triggers pro-inflammatory processes in the early phase of PH development (18, 52). Since B-cell differentiation is stimulated by CD4+ T-cells, their chronic activation leads to PH and increased production of autoantibodies, which are frequently found in PH-associated autoimmune diseases (53, 54). Moreover, the potential of mast cells to activate B-cells through IL-6 signaling has been found to be involved in PH (53). The involvement of NK cells in pulmonary artery wall remodeling has been described in patients with PH, where NK cells significantly upregulated metalloproteinase 9, which in turn affects the functional damage of NK cells themselves (55). Overall, the interplay between different immune cell types during PH development remains unclear and requires further research.

In summary, based on our previous and current results, pulmonary fibroblasts in the adventitia of pulmonary arteries actively regulate the immune response, i.e. proinflammatory polarization of monocytes and T-cells, in their environment through the production of cytokines/chemokines and other molecules and may contribute to local immunomodulation and vessel remodeling in PH. These studies classify PH as another disease in which immunomodulatory approaches may have the potential for novel therapeutic treatment, as has been shown in other pathologies (56–58).

## Data availability statement

The single-cell RNA sequencing data have been submitted to the Gene Expression Omnibus (GEO) repository at the National Center for Biotechnology Information (NCBI). The dataset can be accessed using the accession number GSE234156. To explore the data further, please visit the GEO website at http://www.ncbi.nlm.nih.gov/geo/. 

## Author contributions

Conceptualization: LP-H and KS; Investigation and experiments: LP-H, AB, MK, and JT; scRNAseq analysis: C-JH, TP; Data Analysis: LP-H, AB; Writing the original manuscript: LP-H; KS. Manuscript review and editing: LP-H, AB, KS; Bovine cells provider: ML, HZ; Conditional medium provider from human cells: KH, SC, GK; Funding acquisition: LP-H. LP-H is the guarantor of this work and, as such, has full access to all the data in the study and takes responsibility for the integrity of the data and the accuracy of the data analysis. All authors contributed to the article and approved the submitted version.

## Supplementary material

Supplementary Figure 1 Metabolism and redox status of calf T-cells exposed to LPS/nigericin. **(A)** Glucose fold induced endogenous respiration T-cells exposed to LPS/nigericin for 24 hours, n=4. **(B)** Oxidative phosphorylation activity expressed as endogenous/oligomycin-sensitive respiratory ratio of T-cells exposed to LPS/nigericin for 24 hours, n=3-4. Maximum mitochondrial respiration analysis expressed as uncoupled/oligomycin-sensitive respiration ratio of T-cells exposed to LPS/nigericin for 24 hours, n=3-4. Lactate production expressed as media acidification (ECAR) of T-cells exposed to LPS/nigericin for 24 hours, n=3. P-PDH/PDH protein ratio of T-cells exposed to LPS/nigericin for 24 hours, n=4. **(C)** Quantification of cytosolic ATP of T-cells exposed to LPS/nigericin for 24 hours, n=3. **(D)** Cytosolic redox status expressed as glucose-induced increase in DCF fluorescence in T-cells exposed to LPS/nigericin for 24 hours, n=3.

Supplementary Figure 2 Expressional analysis (mRNA) of cytokines and markers from T-cells exposed to CM-CO/PH Fibs + SAHA. **(A)** Fold change of mRNA expression of IFNγ in bovine CD4+ T-cells exposed to CMCO/PH Fibs+SAHA for early/late time period to CM-CO/PH Fibs, n=3. **(B)** Fold change of mRNA expression of IL4 in bovine CD4+ T-cells exposed to CM-CO/PH Fibs+SAHA for early/late time period to CM-CO/PH Fibs, n=3. **(C)** Fold change of mRNA expression of IL10 in bovine CD4+ Tcells exposed to CM-CO/PH Fibs+SAHA for early/late time period to CM-CO/PH Fibs, n=3. **(D)** Fold change of mRNA expression of TGFβ in bovine CD4+ T-cells exposed to CM-CO/PH Fibs+SAHA for early/late time period to CM-CO/PH Fibs, n=3

## Glossary

**Table:**

CM-CO Fibs	conditioned media from bovine CO Fibs 24-hour cultivation
CM-hCO Fibs	conditioned media from human CO Fibs 24-hour cultivation
CM-PH Fibs	conditioned media from bovine PH Fibs 24-hour cultivation
CM-hPH Fibs	conditioned media from human CO Fibs 24-hour cultivation
CO (h)Fibs	fibroblasts from calf (human) pulmonary arteries of healthy animals
DCs	dendritic cells
dPAs	calf distal pulmonary arteries
ECAR	extracellular acidification rate PH - pulmonary hypertension
IPAH	idiopathic pulmonary artery hypertension
LPS	lipopolysaccharide
NK	natural killers
PASMC	pulmonary artery smooth muscle cells
PAEC	pulmonary artery endothelial cells
PH (h)Fibs	fibroblasts from calf (human) pulmonary arteries of animals with pulmonary arterial hypertension
ROS	reactive oxygen species
TLR4	toll-like receptor 4
