# Supplementary figures and images for: Microenvironmental regulation of T-cells in pulmonary hypertension
Source: Front Immunol. 2023 Jul 11;14:1223122. doi: 10.3389/fimmu.2023.1223122 (PMC10368362; doi:10.3389/fimmu.2023.1223122)

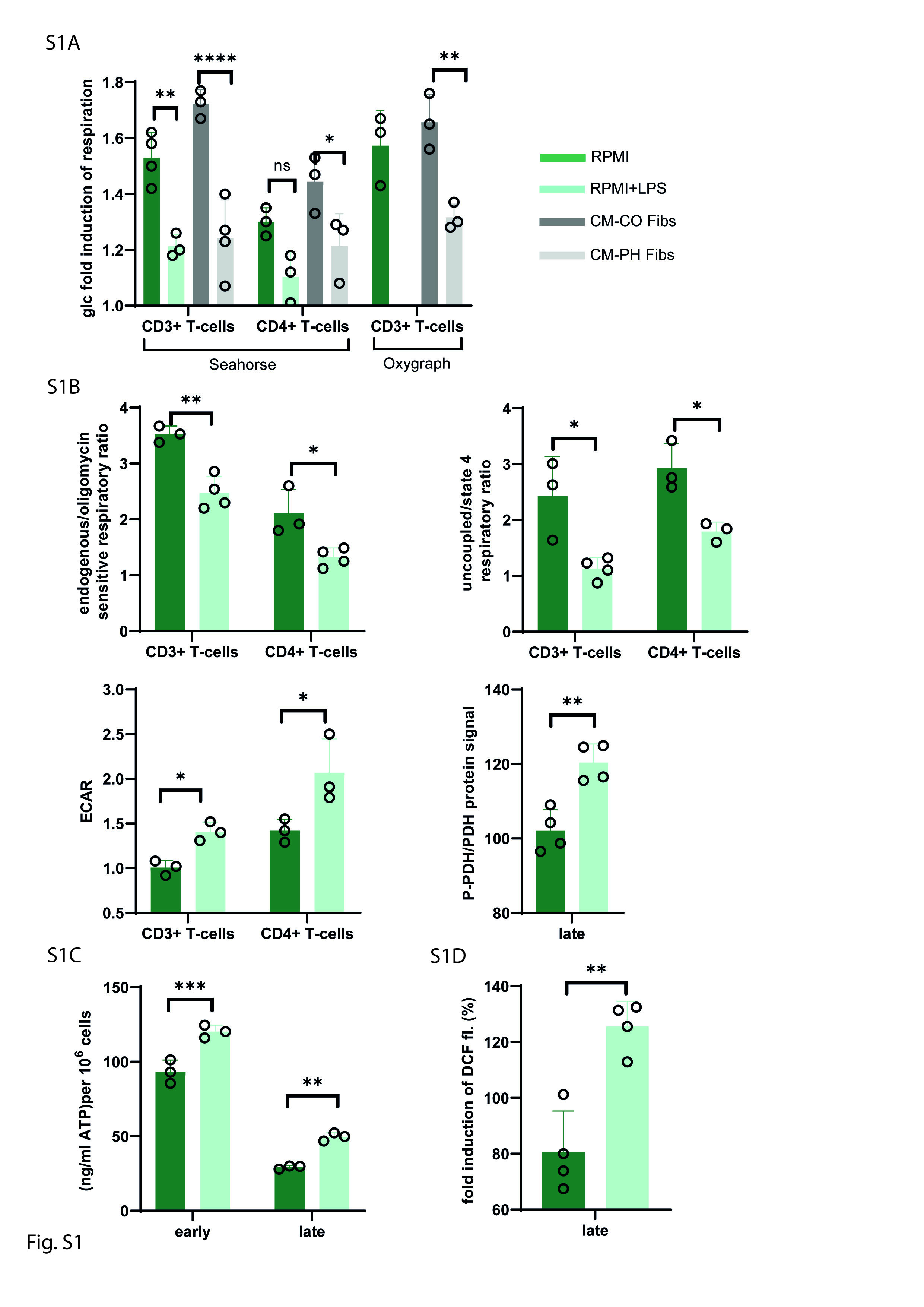

Supplement: Supplementary Figure 1 — Metabolism and redox status of calf T-cells exposed to LPS/nigericin. (A) Glucose fold induced endogenous respiration T-cells exposed to LPS/nigericin for 24 hours, n=4. (B) Oxidative phosphorylation activity expressed as endogenous/oligomycin-sensitive respiratory ratio of T-cells exposed to LPS/nigericin for 24 hours, n=3-4. Maximum mitochondrial respiration analysis expressed as uncoupled/oligomycin-sensitive respiration ratio of T-cells exposed to LPS/nigericin for 24 hours, n=3-4. Lactate production expressed as media acidification (ECAR) of T-cells exposed to LPS/nigericin for 24 hours, n=3. P-PDH/PDH protein ratio of T-cells exposed to LPS/nigericin for 24 hours, n=4. (C) Quantification of cytosolic ATP of T-cells exposed to LPS/nigericin for 24 hours, n=3. (D) Cytosolic redox status expressed as glucose-induced increase in DCF fluorescence in T-cells exposed to LPS/nigericin for 24 hours, n=3. [file Image_1.jpeg]

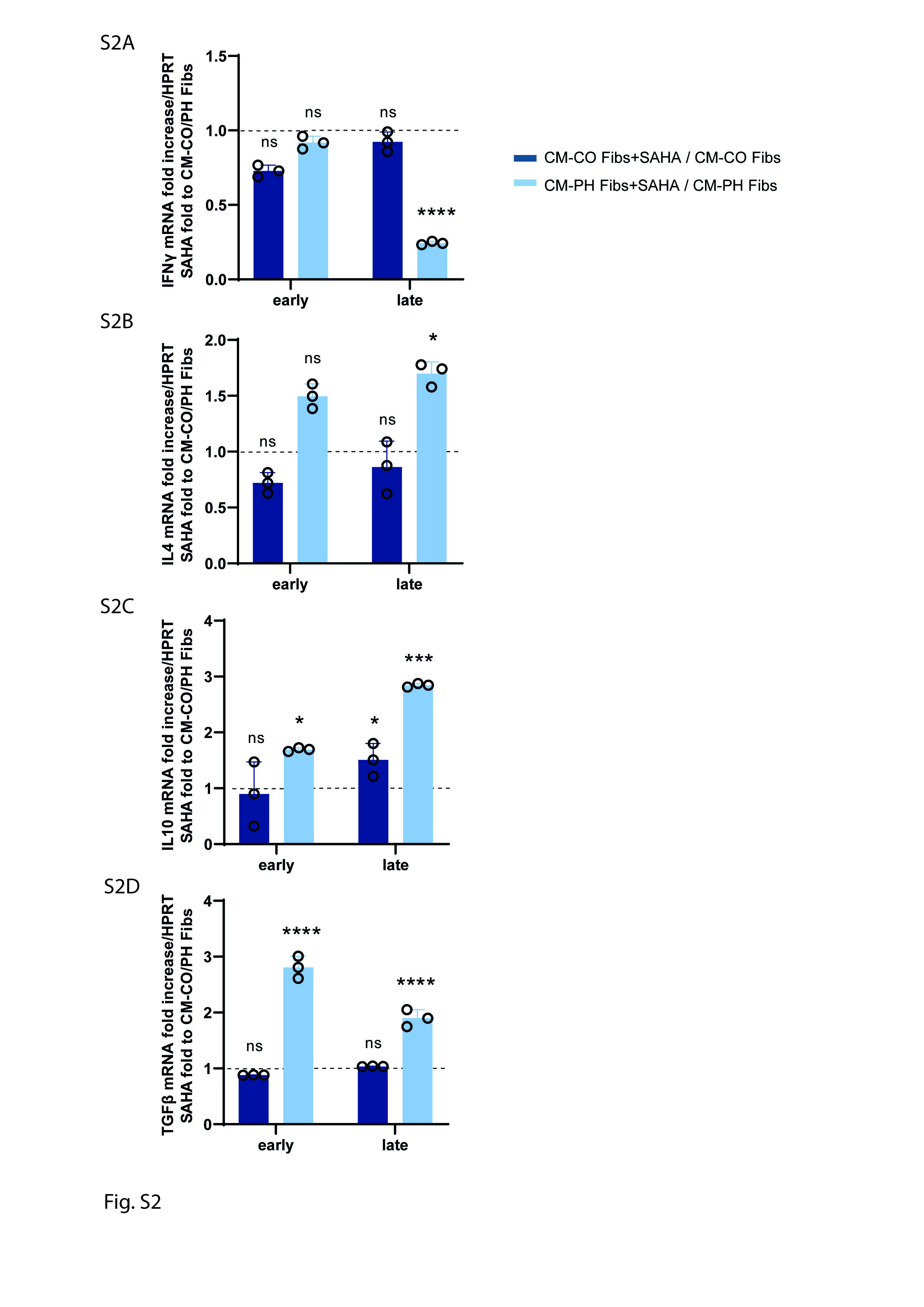

Supplement: Supplementary Figure 2 — Expressional analysis (mRNA) of cytokines and markers from T-cells exposed to CM-CO/PH Fibs + SAHA. (A) Fold change of mRNA expression of IFNγ in bovine CD4+ T-cells exposed to CMCO/PH Fibs+SAHA for early/late time period to CM-CO/PH Fibs, n=3. (B) Fold change of mRNA expression of IL4 in bovine CD4+ T-cells exposed to CM-CO/PH Fibs+SAHA for early/late time period to CM-CO/PH Fibs, n=3. (C) Fold change of mRNA expression of IL10 in bovine CD4+ Tcells exposed to CM-CO/PH Fibs+SAHA for early/late time period to CM-CO/PH Fibs, n=3. (D) Fold change of mRNA expression of TGFβ in bovine CD4+ T-cells exposed to CM-CO/PH Fibs+SAHA for early/late time period to CM-CO/PH Fibs, n=3 [file Image_2.jpeg]
